# Why Is There So Much More Research on Vision Than on Any Other Sensory Modality?

**DOI:** 10.3389/fpsyg.2019.02246

**Published:** 2019-10-04

**Authors:** Fabian Hutmacher

**Affiliations:** Department of Psychology, University of Regensburg, Regensburg, Germany

**Keywords:** visual dominance, visuo-centrism, visual turn, social constructionism, history of the senses, multimodal integration, perception

## Abstract

Why is there so much more research on vision than on any other sensory modality? There is a seemingly easy answer to this question: It is because vision is our most important and most complex sense. Although there are arguments in favor of this explanation, it can be challenged in two ways: by showing that the arguments regarding the importance and complexity of vision are debatable and by demonstrating that there are other aspects that need to be taken into account. Here, I argue that the explanation is debatable, as there are various ways of defining “importance” and “complexity” and, as there is no clear consensus that vision is indeed the most important and most complex of our senses. Hence, I propose two additional explanations: According to the methodological-structural explanation, there is more research on vision because the available, present-day technology is better suited for studying vision than for studying other modalities – an advantage which most likely is the result of an initial bias toward vision, which reinforces itself. Possible reasons for such an initial bias are discussed. The cultural explanation emphasizes that the dominance of the visual is not an unchangeable constant, but rather the result of the way our societies are designed and thus heavily influenced by human decision-making. As it turns out, there is no universal hierarchy of the senses, but great historical and cross-cultural variation. Realizing that the dominance of the visual is socially and culturally reinforced and not simply a law of nature, gives us the opportunity to take a step back and to think about the kind of sensory environments we want to create and about the kinds of theories that need to be developed in research.

## Introduction

**FIGURE 1:**
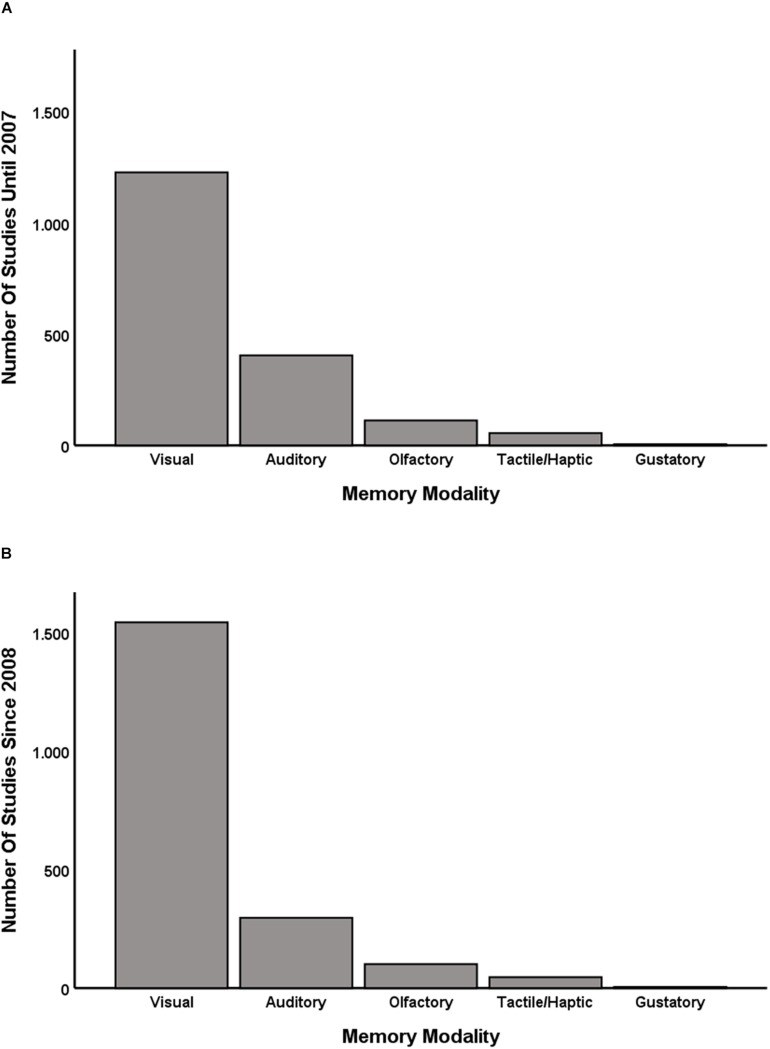
The number of studies on the different sensory modalities published until the end of 2007 is depicted in panel **(A)**; the number of studies on the different sensory modalities published since the beginning of 2008 is depicted in panel **(B)**. Following the procedure by Gallace and Spence (2009), I searched the PsycINFO database for studies containing “auditory,” “gustatory,” “olfactory,” and “tactile/haptic memory” in the title (search date: May 29, 2019) (see Supplementary Data Sheet 1).

It has already been observed, about a 100 years ago, that research on perception and perceptual memory often in fact is research on *visual* perception and *visual* memory, while other sensory modalities play a minor role (Katz, 1925/1989). Gallace and Spence (2009) supported this observation with empirical data. When searching the PsycINFO database for studies containing “visual,” “auditory,” “gustatory,” “olfactory” or “tactile/haptic memory” in the title, they found two interesting results. First, there were more studies on visual memory than studies on the memory of all other sensory modalities combined. Second, while there were still a considerable number of studies on auditory memory, research on olfactory, gustatory, and haptic memory was even more limited. I repeated the same search for this paper (see Figure 1A). As Gallace and Spence (2009) conducted their search more than 10 years ago, I also added the data for the past decade (see Figure 1B; note that an updated version of the graph containing data until the end of 2010 is presented by the authors themselves in Gallace and Spence, 2014, p. 112). The pattern has remained the same. If anything, the proportion of studies on visual memory has increased (from 68.04% among the studies conducted until 2007 to 77.46% among the studies conducted since the beginning of 2008).^1^

The question is: Why? Why is there so much more research on vision than on any other sensory modality? There is a seemingly easy answer to this question, which I will call the “textbook explanation”: Vision is our most important and most complex sensory modality and this is mirrored in the number of studies. Although there are indeed arguments in favor of the textbook explanation, I will show that this answer alone is too simplistic for at least two reasons. First, the textbook explanation is debatable insofar as the notion that vision is our most important and most complex sensory modality depends on the definition of “importance” and “complexity.” Second, the textbook explanation is incomplete as there are further explanations to be considered: (a) the idea that the dominance of the visual has methodological-structural reasons, and (b) the observation that the importance ascribed to the different sensory modalities varies across times and cultures. Hence, the impression that vision is our most important sensory modality flows partially, at least, from the fact that contemporary Western societies are visual societies and that researchers from these countries still dominate the scientific discourse in psychology.

## The Textbook Explanation

If you open a textbook on perception or cognitive psychology, you will realize that normally more chapters are dedicated to vision than to any other modality (e.g., Kandel et al., 2000; Goldstein, 2010; Sternberg and Sternberg, 2017). Apparently, this decision seems self-evident to the authors and is thus often either not explained at all or explained only briefly. Sternberg and Sternberg (2017) simply state, for instance, that “vision is the most widely recognized and the most widely studied perceptual modality” (p. 72) while Kandel et al. (2000) claim that “[m]ost of our impressions about the world and our memories of it are based on sight” (p. 492). In the same spirit, Gerrig and Zimbardo (2008) write that “[v]ision it the most complex, highly developed, and important sense for humans and most other mobile creatures” (p. 103). A more detailed explanation of this idea can be found in a chapter on perception by Pike et al. (2012):

[That there has been far more research on vision] is because when we interact with the world we rely more on vision than on our other senses. As a result, far more of the primate brain is engaged in processing visual information than in processing information from any of the other senses (p. 67).

The last two quotes contain the two elements of what I will call the “textbook explanation.” According to the textbook explanation, there is so much research on vision compared to research on the other modalities (1) because vision is more *important* than other modalities for our daily experience as well as the way we interact with the world and (2) because the processing of visual information is far more *complex* and occupies larger parts of our brain than the processing of sensory information from other modalities.

In fact, a wide range of evidence supports these two claims. As far as the *importance* of vision is concerned, two kinds of importance have to be distinguished: the *subjective* importance, that is the importance of a certain sensory modality from a first-person perspective, and the *empirical* importance, that is the importance of a certain sensory modality when it comes to processing and remembering information as well as navigating through the world. Let us examine both and let us begin with the subjective importance.

**FIGURE 2:**
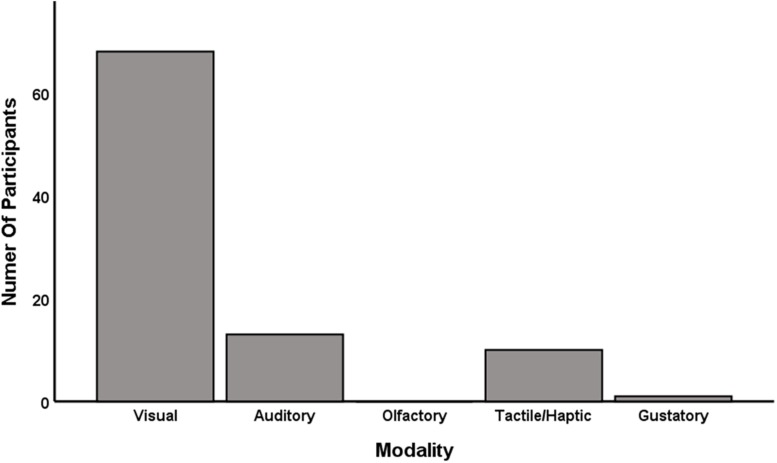
Participants of an online-survey (*N* = 91) were asked to answer the following question: “Losing which modality does scare you the most?” The response options were presented in random order (see Supplementary Data Sheet 2).

When asked to rate the extent to which different modalities are part of the experience with objects, most people put vision first (Tranel et al., 1997; Schifferstein, 2006; Schifferstein et al., 2010). Although these studies indicate that vision is indeed the most important modality for most people from a subjective point of view, one can address this question even more directly: Imagine losing one of your sensory modalities. Losing which modality does scare you the most? I conducted a survey asking exactly this question (*N* = 91, 63 females, 27 males, 1 diverse, age 19–62 years, *M*_*age*_ = 29.44, *SD*_*age*_ = 10.96). For the overwhelming majority of people the answer is *vision* (73.63%). The pattern across the different modalities (see Figure 2) is similar to the pattern for the number of studies conducted in each sensory modality (see above, Figure 1). This similarity suggests a straightforward answer to the question as to why there is so much more research on vision than on any other sensory modality: People tend to investigate those modalities that are most important to them. Interestingly, the subjective dominance of the visual is also reflected in language. As demonstrated by Winter et al. (2018), there is a higher frequency of visual words and a greater number of unique visual words compared to words for other sensory modalities in a wide range of English corpora. As it has been argued that the vocabulary of a language is optimized for satisfying the communicative needs of their speakers (for a discussion of this idea in the context of colors see e.g., Berlin and Kay, 1969; Cook et al., 2005), these results seem to mirror the subjective importance of the visual.

The idea that vision is indeed the most important modality is supported by numerous studies demonstrating *visual dominance* (for a meta-analysis, see Hirst et al., 2018; for a recent philosophical account, see Stokes and Biggs, 2014). The term “visual dominance” refers to the observation that information from different senses is not treated equally. Rather, the processing of visual information seems to dominate the processing of information from other modalities. The reasons for this are still debated. While some argue that visual dominance flows from the fact that vision is more accurate and reliable than the other senses, others argue that the opposite is true and that people have to focus on visual input, because of its rather weak capacity to alert the organism to its occurrence (see Posner et al., 1976, for the classic and Spence et al., 2001, for an updated elaboration of the latter view). One famous example of visual dominance is the so-called “Colavita effect” (Colavita, 1974; Colavita et al., 1976; for a recent review, see Spence et al., 2012). When a visual and an auditory stimulus are presented simultaneously, participants show a strong tendency to respond to the visual stimulus. Even more, participants frequently report not having perceived the auditory stimulus at all. The finding of visual dominance over auditory stimuli has also been extended to visual dominance over haptic stimuli (Hecht and Reiner, 2009; for earlier attempts, see Rock and Victor, 1964; Rock and Harris, 1967).

Hence, evaluating both the subjective as well as the empirical importance of the different sensory modalities seems to lead to the conclusion that vision is more important than the other modalities. However, what about complexity? The core idea of the complexity argument is simple: A large part of the human – or more generally speaking: primate – neocortex is involved in processing visual information, while information from other sensory modalities is processed in far smaller brain regions. When investigating the macaque neocortex, for instance, it turned out that 54% of the macaque neocortex are involved in visual processing (Van Essen et al., 1990). In contrast, only a small fraction of the neocortex is exclusively dedicated to auditory (3%) or somatosensory processing (11%). To this, it can be added that the estimated number of sensors and afferents as well as the information transmission rate is higher for vision than for any other sensory modality (see Zimmerman, 1989). Thus, one may argue that the greater “brain power” available for processing visual information allows for a more fine-grained analysis of the incoming visual stimulation compared to the stimulation from other modalities.

## Questioning the Textbook Explanation

The textbook explanation can be challenged in two ways: by showing that the arguments regarding the importance and complexity of vision are debatable and by demonstrating that the textbook explanation is *incomplete* as there are other aspects that need to be considered.

### Why the Textbook Explanation Is Debatable: Importance

At first glance, it appears obvious why the vast majority of participants, in the survey reported above, stated that they are most afraid of losing their visual abilities. Just imagine for one moment, how vital vision is for most of your daily activities, ranging from hobbies (e.g., reading, watching a movie, playing tennis) and daily routines (e.g., grocery shopping, going by car or bike, cleaning your apartment) to your work environment (e.g., writing mails, working on your computer, administering any kind of machine). However, our present-day societies offer a wide range of support to blind people so that they can remain active members of their community. Although losing sight may be perceived as a traumatic event and although it profoundly changes the way one interacts with the world, it does normally not endanger the survival of the individual – and probably not even its social integration.

Now imagine – for comparison – losing your haptic abilities: You would not feel anything when hugging your loved ones or when caressing their faces; you could not tell whether your back hurts or whether you are comfortably seated; you would not notice when stepping barefoot on a piece of broken glass (unless you see the blood coming from you wound), and so on. In short, you would be deprived of some of the most important and most intimate aspects that come with the fact that human beings are physical beings that are – literally – *in touch with the world*, not to forget that losing the sense of touch would drastically diminish the ability to detect dangers for the physical integrity. In this context, it is interesting to consider congenital insensitivity to pain, a very rare condition in which people are – as the name already suggests – insensitive to pain from birth onward (for a review, see Nagasako et al., 2003). Although these people have no cognitive defects, they often die in childhood and generally have a decreased life expectancy. It is easy to see why:

[These people are not] able to determine if a bone is broken or if they have bitten off the tip of their tongue unless they see the swelling of the surrounding tissue or taste blood in their mouth. Because of this inability to sense pain, it is common for patients with congenital insensitivity to pain to have unseen infections as well as have a multitude of bruises, wounds, and broken bones over their lifetime (Hellier, 2016, p. 118).

Thus, it seems that – at least in our present-day societies – haptic abilities such as the ability to sense pain are more important *for our survival* than visual abilities. In line with this, it has also been hypothesized that physical contact is a necessary precondition for the healthy development of the individual. Skin-to-skin contact between the mother and the infant in the first hour after birth has vital advantages for short- and long-term health (Klaus et al., 1972; for a recent review, see Widström et al., 2019). As this effect may rather be due to the formation of an emotional bond which is facilitated through skin-to-skin contact rather than due to the skin-to-skin contact *per se*, it is interesting to consider another developmental issue: At birth, our visual system is severely underdeveloped. Hence, in the first months of their lives, newborns need to learn to make sense of the incoming visual information. As the sense of touch already plays a crucial role for the unborn child, it seems legitimate so speculate that newborns use their well-developed sense of touch to achieve this (see Martin, 1992; Grunwald, 2017). In fact, it has been demonstrated that newborns can extract the shape of an object by haptically exploring it and that they can transfer this knowledge so that they are able to visually recognize the same object they had only touched before (Streri and Gentaz, 2003, 2004; Sann and Streri, 2007). In addition, remember that many of the newborn reflexes such as the grasp or suck reflex are shown as a response to being touched. From this perspective, one may argue that our sense of touch is more important than our sense of vision as the former plays an essential role in the development of the latter (see e.g., Gottlieb, 1971, for an ontogenetic account of sensory function). In particular, it has been argued that “early tactile experiences […] might strongly contribute to shaping and characterizing the emotional, relational, cognitive, and neural functioning of the adult individual” (Gallace and Spence, 2014, p. 178).

Note, that these remarks about the importance of haptics are not meant to establish a haptic-centered version of the textbook explanation claiming that haptics should be treated as the most important sensory modality. Rather, I have tried to show that it is far more difficult to decide which sensory modality is most important for human beings than one may at first think. In fact, it is a matter of perspective; it is a matter of the aspects taken into consideration (for an example of differences between people, see Delwiche, 2003). Interestingly, the reported differences between vision and haptics may be the result of the fact that vision is a *distant sense* while touch is a *proximal sense* (for this distinction see e.g., Katz, 1925/1989; Rodaway, 1994; Trope and Liberman, 2010; Klatzky and Lederman, 2011).^2^ Expressed in plain words, touch gives us information about the way our body is embedded in the environment. Touch is an integral part of the existential experience of being a physical creature (see e.g., O’Shaughnessy, 1989). At the same time, we are consciously unaware of most of our haptic sensations: You can direct your attention toward your feet in order to find out how they feel in your shoes right now. If you are not intentionally directing your attention toward your haptic sensations, however, you will not notice them most of the time (until someone taps you on your shoulder or you take a hot shower on a cold winter day). Compare this to vision: As long as you are awake, it is hard to prevent the visual impressions and changes in your environment from entering your consciousness – no matter how relevant or irrelevant this information may be for your current goals.^3^ That is because vision as a distant sense informs us about the surroundings; it informs us *about the world*. Thus, vision is especially important when it comes to actively exploring and navigating in this world:

A view comprehends many things juxtaposed, as co-existent parts of one field of vision. It does so in an instant: as in a flash one glance, an opening of the eyes, discloses a world of co-present qualities spread out in space, ranged in depth, continuing into indefinite distance […] (Jonas, 1954, p. 507).

Put differently, vision as a distant sense has a *qualitatively* different function than touch as a proximal sense. This qualitative difference renders conclusions about the absolute importance of a given sensory modality almost impossible. In this context, one may additionally consider olfaction: “Often, we rely on our sense of smell in order to decide whether or not it is safe to engage further with a given stimulus” (Gallace et al., 2012, p. 16). Thus, although smell may play a rather minor role in everyday life, it becomes extremely important in potentially harmful or even life-threatening situations, such as determining whether some food is rotten, detecting a gas leak or smelling fire.

In short, the degree to which vision dominates the research on the different sensory modalities cannot simply be explained by claiming that vision is the most important modality. As it will turn out in the next section, the same applies to the complexity argument.

### Why the Textbook Explanation Is Debatable: Complexity

The complexity argument was based on the assumption that a large part of the human brain is specialized on visual processing while relatively small parts are specialized on processing information from other sensory modalitities. This assumption has been questioned in recent years: Instead of regarding the senses as strictly separated entities, it has become quite common to accept that they often interact and influence each other, which is also mirrored in the neural underpinnings (for reviews see e.g., Ghazanfar and Schroeder, 2006; Alais et al., 2010). It has even been argued that the multisensory nature of the neocortex may force us “to abandon the notion that the senses ever operate independently during real-world cognition” (Ghazanfar and Schroeder, 2006, p. 278). Interestingly, multisensory integration does not only occur in the later brain regions in the temporal and frontal cortices, but also in earlier brain regions and even in the primary sensory cortices. Moreover, brain regions previously believed to be visual by nature are used during Braille reading (e.g., Sadato et al., 1996; Büchel et al., 1998) and for processing auditory information (e.g., Burton et al., 2002; Röder et al., 2002) in blind people. As multisensory processing appears to be the rule rather than the exception, claiming that a large part of the human brain is exclusively specialized for processing visual information seems at least debatable (see e.g., Shimojo and Shams, 2001) – or as Lacey and Sathian (2008) put it: “The crossmodal activity of visual cortex likely reflects modality-independent representations of objects and other stimuli such as motion […]. Such findings increase support for the idea of a ‘metamodal’ brain organized around task processing rather than separate sensory streams” (p. 257).

However, this line of reasoning is not the only way to question the complexity argument: Why should the size of brain regions specialized on processing information from a certain modality be the only criterion at all, when it comes to determining complexity? One could also take into account the number of different receptor cells, for instance: While humans have only two major classes of photoreceptor cells (rods and three kinds of cones), they possess several hundred different kinds of olfactory receptor cells (Axel, 1995; Glusman et al., 2001) and can discriminate more than one trillion olfactory stimuli (Bushdid et al., 2014). Alternatively, remember that the skin is the largest sensory organ of the human body, accounting for more than a tenth of total body weight (Montagu, 1971; Field, 2001; Martini and Nath, 2009). Again, these examples are not meant to claim that vision is *definitely not* the most complex modality, but rather that there are various ways of defining complexity (for an attempt to distinguish different meanings of complexity in the chemical senses, see Spence and Wang, 2018). Moreover, no definition presented here seems to provide clear evidence that vision is beyond any doubt the most complex sensory modality. As both the importance and complexity argument are insufficient for explaining the degree to which vision dominates the research on the different sensory modalities, it seems necessary to look for other possible explanations.

## Alternative Explanations

Here, I present and discuss two additional explanations which can help illuminating the bias toward vision in research. The methodological-structural explanation claims that research on vision is often easier than research on other modalities and that this the result of an initial bias toward vision that reinforces itself; the cultural explanation carves out that the dominance of the visual is not a historical constant, but rather a result of the way (Western) societies are designed.

### The Methodological-Structural Explanation

Imagine having to set up an experiment that investigates long-term memory for everyday objects. If you decided to present the objects *visually* on a computer screen, your task would be straightforward: Use your favorite search engine and collect as many images of as many different objects as possible. Instead, you may also refer to one of the publicly available databases, offering thousands and thousands of images (see e.g., Brady et al., 2008; Konkle et al., 2010). If you decided to present the objects *haptically*, your task would be much harder: Even if you had a list containing the names of all objects used in a previous study as well as photos of these objects (e.g., Hutmacher and Kuhbandner, 2018), this would be of limited use for your own study, as it would not free you from the necessity to buy and collect all the objects on your own. And the struggle continues: The objects gathered for haptic presentation will occupy much more space than the images of objects stored on your hard drive. Even setting up the actual experiment is easier when working with images presented on a computer screen, as with all programs designed for creating experiments, many potential methodological flaws are easy to avoid. The duration of the stimulus presentation can be determined precisely, counterbalancing within and between participants is normally achieved with a couple of mouse clicks (or lines of code), and the responses of the participants are automatically recorded and coded as correct or false. All these things become vastly more difficult when doing the same experiment involving haptic exploration, as the experimenter has to navigate carefully between the objects (a wine glass *is* fragile, the image of a wine glass is not), keep track of the objects that were already presented, make sure that the participants do not explore the objects too long, and so on.

In short, while there is a lot of off-the-shelf technology available for studying vision, this is not the case for other sensory modalities such as touch (see e.g., Krueger, 1989, p. 2, reporting personal communication from Lederman). However, this conclusion is not the end of the methodological-structural explanation. Instead, one could ask further: What could be the reason that the available technology is better suited for studying vision than the other modalities? There are two possible answers to this question.

First, one may argue that vision is *by nature* easier or that the other senses are *by nature* harder to investigate. Maybe vision has a subtle advantage as it “is the ideal distance-sense” (Jonas, 1954, p. 517), that is, as it does not only *allow for distance* to the stimulus (light travels farther than sound or smell), but *gains by distance* as “the best view is by no means the closest view” (p. 518) – a feature, which is perfectly suited for the distanced and objective perspective of an experimenter (see Classen, 1993). In contrast, it seems hard to imagine how there could be off-the shelf technology for studying the chemical senses, for instance: Although researchers have tried to, no one has yet found a digital way of stimulating the chemical senses, which would be an important precondition for setting up standardized and easily controllable experiments (see Spence et al., 2017). The same can be said about haptic long-term memory: Whoever wants to study the haptic exploration of everyday objects will have to collect the respective objects. There seems to be no way around this.

Even if there is a way around this in some cases, however, the tools developed to study other senses such as touch (see e.g., Grunwald et al., 2002; Mueller et al., 2014), are not widely spread and were constructed by the authors in a laborious process in order to test their hypotheses (see Grunwald, 2017). Rather than demonstrating that present-day technology used to investigate haptics is equal to the technology to investigate vision, these efforts to create adequate instruments in the absence of an established technology remind of the situation at the end of the 19th century, when the first experimental psychological laboratories were founded (see e.g., Caudle, 1983; Schoenherr, 2017). At that time, creating tools for research on vision was a laborious process. Hence, one can get the impression that the development of haptic technology lags behind in time.

This observation leads to the second answer to the question as to why the available technology is better suited for studying vision than the other modalities: The “Matthew effect” (Merton, 1968) describes the fact that the networks of science are designed in a way that creates more attention for (and allocates more rewards to) already well-known researchers and well-established research topics while rather unknown fields and scientists remain largely unnoticed. Thus, one could hypothesize that instead of (or at least in addition to) being naturally better suited for investigation, vision may have had an arbitrary advantage in the beginning of experimental research and that this initial advantage has perpetuated and possibly even reinforced itself since then. Classen et al. (1994) confirm the idea of a Matthew effect in the research on different modalities by comparing the status of vision to the status of smell: “While the high status of sight in the West makes it possible for studies on vision and visuality […] to be taken seriously, any attempt to examine smell runs the risk of being brushed off as frivolous and irrelevant” (p. 5). Expressed differently, funding for research into vision might be much easier to obtain than funding for smell or touch. This might in turn bias researches toward doing research on vision as it is easier to get funding, and so on.

There are various possible reasons why the study of vision may have had an advantage in the beginning of experimental research: (1) researchers at the time may have had personal reasons to study vision instead of other modalities (e.g., because they had the subjective impression that vision is their most important modality); (2) researchers may have been biased toward vision due to a long history of visual dominance in Western societies (see the next section); (3) apparently, “early psychologists enthusiastically borrowed and adapted the scientific instruments that had heretofore been used to explore problems related to the physical laws of acoustics and optics and the physiology of the sense organs” (Caudle, 1983, p. 20–21), almost automatically leading to research focusing on vision and hearing;^4^ (4) vision may have a special appeal to scientists as it appears more objective to modern scientists following empiricist traditions than the other senses (see above): “The detachment of sight, distancing spectator from spectacle, makes the cherished objectivity of the scientist possible” (Classen, 1993, p 6).

In conclusion, the methodological-structural explanation claims that there is more research on vision because the available, present-day technology is better suited for studying vision than for studying other modalities. Although one may claim that vision is easier to investigate by nature, it seems quite likely that this claim and thus the technological advantage for vision is at least partially the result of a Matthew effect: As there is more research on and easier accessible technology for vision compared to other modalities today, there will most likely be more research on and technology for vision tomorrow. In addition to the self-perpetuating process proposed by the Matthew effect, there may also be a cultural explanation for the bias toward vision.

### The Cultural Explanation

At first sight, one may think of visual dominance as a cultural constant that can be traced back to antiquity (for a history of the senses, see e.g., Classen, 1993; Jütte, 2005). When Plato writes about the senses, for instance, he puts the greatest emphasis on vision, which he even describes as “divine” in one place (see Jütte, 2005, p. 35). Generally speaking, he favors the supposedly more rational “higher senses” vision and audition over the other “lower senses,” which he believed to be more subjective and bound to bodily reactions (see e.g., Schellekens, 2009). In a similar manner, Aristotle creates a ranking of the senses, putting vision first, followed by hearing, smell, taste, and touch. Although subsequent philosophers did not agree with the classical Aristotelian hierarchy in every respect, vision is almost always ranked as the highest sense in Western societies throughout the medieval ages up until today. In this context, it is especially interesting that the study on the frequency of words referring to the different sensory modalities quoted above, did not only find an overall higher frequency of visual words in the investigated English corpora. When looking at the average frequencies for each modality based on the ten most exclusive words per modality, there was hardly any change in the past 200 years (see Winter et al., 2018, Figure 5), suggesting that the hierarchy of the senses remained unchanged.

Although the Aristotelian hierarchy has undeniably had a huge influence on the conceptualizations of generation upon generation of philosophers and although one may argue that there is a long history of visual dominance, matters become vastly more complicated when taking a closer look at the available sources. First, although positioning vision first in his hierarchy, Aristotle also states that the sense of touch is “much more closely related than the other senses to the four elements, since the properties of the elements (dry, wet, cold, warm) are all palpable,” leading him to the conclusion “that without the sense of touch there could be no other senses” (Jütte, 2005, p. 42) – an idea Thomas Aquinas agreed with more than a 1000 years later. Thus, one could claim that the hierarchy proclaimed by Aristotle was not meant to be interpreted that strictly after all. This idea is in line with Avicenna’s view on Aristotle. As the medieval, Persian polymath “understood it, what Aristotle meant was that with respect to honor the primacy of the sense of sight applied, but that from the point of view of natural aptitude the sense of touch merited priority” (Jütte, 2005, p. 69). Second, it can be demonstrated that the dominance of the visual – supposedly already existing in the times of Aristotle – was less pronounced for a long time, that is, that the non-visual senses have lost ground against sight in the course of the past centuries. Hence, rather than being a cultural constant, visual dominance turns out to be heavily influenced by human decision-making. I will illustrate this idea using three different examples.

First, consider the shift from an oral, hearing-dominated to a written, sight-dominated culture (e.g., McLuhan, 1962; Ong, 1967), which was a result of the “Gutenberg Revolution.” While knowledge was predominantly transmitted orally before the invention of the printing press, vision has become the common means of acquiring information since then. Note, that this shift from hearing to sight arguably also changed interactions between people: The oral transmission of knowledge – and of literature, by the way – requires at least two people (a teacher and a student; someone who is telling a story and someone who is listening to it); in contrast, reading a book does not require any personal interaction – you can do it entirely on your own.

Second, take the decrease of the importance of smell. As Classen (1993) points out, “[i]n the pre-modern West […], smell was associated with essence and spiritual truth, while sight was often deemed a ‘superficial’ sense, revealing only exteriors” (p. 7). Moreover, the strength of the odor of a plant was associated with its presumed medical power: In order to protect themselves against epidemic diseases such as the plague, people in the medieval ages often carried a pomander with them, as they believed that strong scents are an antidote against the odors of illness which were considered to be the cause of infection. The idea that scents are important was also mirrored in the design of monastery gardens of the time: Flowers were mostly grown for practical purposes, that is for cooking or producing medicine – and as their scent rather than their visual appearance was considered to carry its potency, they “were grown together with garlic, onions and other herbs and vegetables used in cooking” (p. 22). This slowly changed from the 16th and even more so from the 18th century onward: As the belief in the healing power of scents faded away and as gardens were also cultivated for aesthetical and recreational reasons, visuals became increasingly more important.^5^ In a similar spirit, it has recently been argued that the idea that humans have a poor sense of smell “derives not from empirical studies of human olfaction but from a famous 19th-century anatomist’s [Paul Broca] hypothesis that the evolution of human free will required a reduction in the proportional size of the brain’s olfactory bulb” (McGann, 2017, p. 1). In contrast to this hypothesis, it has been shown that the olfactory abilities of humans are in fact quite good.

Third, imagine walking through a modern museum exhibiting sculptures: You would probably not in your wildest dreams think of touching these sculptures – and if you did, security guards, alarms, not to-be-crossed lines on the floor or transparent cases around the sculptures would remind you immediately that art is not to be touched (see Gallace and Spence, 2014, for the few contemporary counterexamples). In contrast to this, it has been observed that in medieval culture, sculptures were “far more publicly accessible” (Jung, 2010, p. 215). One may even say that sculptures were *meant* to be touched: “[M]edieval people stroked, held, and cradled sculptural representations” (Griffiths and Starkey, 2018, p. 9). Note, that remnants of these haptic worshipping traditions have survived until today: The right foot of the bronze statue of St. Peter in the St. Peter’s Basilica in Rome is worn down by pilgrims who have touched and kissed it for centuries, for instance (for a description of similar rituals, see Frijhoff, 1993).

As these three examples demonstrate, one can trace an ongoing shift toward vision throughout history. However, the bias toward vision may be even more pronounced in our present-day societies than ever before: Beginning with the invention of movies, cinema, and television and even more so in the face of the omnipresence of smartphones and computers, visual technologies increasingly regulate our daily lives:

Modern life takes place onscreen. […] Human experience is now more visual and visualized than ever before from the satellite picture to medical images of the interior of the human body. […] In this swirl of imagery, seeing is much more than believing. It is not just a part of everyday life, it is everyday life (Mirzoeff, 1999, p. 1).

To give one illustrative example, consider the now-common habit of taking a picture of your meal and of sharing it on social media before starting to eat. It has been hypothesized that this habit has profoundly changed the way restaurants are recommended. While recommendations used to be based on the opinion of friends and colleagues or on written reports in newspapers, magazines or reference guides like the famous “Guide Michelin,” they are now increasingly based on the visual appearance of the food. This may ultimately lead chefs and restaurant owners to pay more attention to the visual arrangement of the food they serve, or even to prepare the food in a way that is going to look good on Instagram (see e.g., Saner, 2015; Spence et al., 2016; Lee, 2017; for an experimental investigation of the importance of the orientation in the plating of food, see Michel et al., 2015). More broadly speaking, paying attention to visual aspects seems crucial to achieve important goals in life such as finding a job or a partner as well as improving social relationships – just think of the importance of visuals when presenting oneself on an online dating website, sharing holiday pictures on social media or applying for a job with a well-designed resume. In accordance with these kind of phenomena, the necessity of a “visual” or “pictorial turn” has been proclaimed in the cultural sciences (see e.g., Boehm, 1994; Mitchell, 1994; Boehm and Mitchell, 2009; Alloa, 2016). Such a visual turn can supposedly have a double function: it can account for the dominance of the visual by emphasizing the importance of research on the topic and it can help to create both an appropriate methodology to investigate and appropriate theories to describe the visual turn.

Overall, it seems that the dominance of the visual is not a cultural constant. It should not be forgotten, however, that everything that has been discussed so far primarily referred to – pre-modern, modern, and postmodern – *Western* cultures and societies. As it will turn out, considering non-Western societies only confirms the ideas presented so far: The dominance of the visual is at least partially the result of human decision-making and should thus not be regarded as an unvarying historical constant. Two examples shall suffice to illustrate the enormous cross-cultural variability.

First, a recent study has demonstrated that there is no universal hierarchy of the senses by investigating 20 different languages including three unrelated sign languages (Majid et al., 2018). The authors created stimulus sets for each of the five Aristotelian sensory modalities and asked their participants to describe them (What color is this? What sound is this?) in order to find out how detailed these stimuli are coded in each language. Apart from the fact that smell is poorly coded in most languages, there was no common hierarchy of the senses. While English indeed seems to have a visual bias (see the study by Winter et al., 2018, discussed above), other languages seem to have a gustatory bias (e.g., Turkish and Farsi) or a bias toward touch (e.g., Dogul Dom spoken in Mali and Siwu spoken in Ghana). Thus, the authors conclude, “that the mapping of language into senses is culturally relative” (p. 11374) and that “either by cultural tradition or by ecological adaptation, each language has come to concentrate its efforts on particular sensory domains” (p. 11375).

Second, let us examine one of the cultures for which sound seems to be more important than vision: the culture of the Songhay of Niger. It is important to note, that for them, sound is not only important because like in any oral culture, knowledge is transmitted by spoken words, but because the sounds of the words *themselves* are believed to carry energy and power:

[The Songhay] believe that sound, being an existential phenomenon in and of itself, can be the carrier of powerful forces. […] We take the sound of language for granted. [The Songhay] consider language […] as an embodiment of sound which practitioners can use to bring rain to a parched village or to maim or kill their enemies (Stoller, 1984, p. 569).

Thus, for the Songhay, “signifiers can function independently of their signifieds” (Howes, 1991, p. 10), that is, the meaning of a word and its sound can be separated and perform different functions.

What can we learn from considering the cultural explanation regarding the question why there is so much more research on vision than on any other sensory modality? The answer is quite simple: Living in a visual society means living in a society placing high value on vision and comparably little value on the other senses – a tendency that is mirrored in the number of studies on vision. Put differently, a society placing higher value on the other senses would probably develop more balanced research agendas (i.e., research agendas in which the bias toward vision would be less pronounced).

## Conclusion: Living in a Visual Society and the Need for Integration

Why is there so much more research on vision than on any other sensory modality? This paper has discussed three different explanations. The only explanation that can be found in contemporary books on perception and cognitive psychology, which I have called the textbook explanation, claims that the reason for the bias toward vision is its importance and complexity. Although there are arguments in favor of this explanation, the validity of these arguments seems debatable as it crucially depends on the definition of importance and complexity. Apart from that, the textbook explanation is at least incomplete as there are other aspects that need to be taken into consideration. As the methodological-structural explanation proposes, the present-day technology is better suited for studying vision than for studying other modalities, which may be the result of a Matthew effect reinforcing the advantage of the visual. In addition to that, the cultural explanation suggests that the dominance of the visual is not an historical constant, neither in Western nor in non-Western societies, and should consequently be viewed as being influenced by human decision-making. In my opinion, there a two important lessons to be learned from this outcome: the necessity of diversity and the necessity of integration. Let us consider both.

First, the necessity of diversity: Diversity is not necessarily good *per se*. In the event that there was in fact one modality, which is much more important and complex than all the others, a research bias toward this modality would be perfectly understandable (and a visual turn advisable). As “[a]ny classification of the senses is first and foremost an analytical device, a simplification and an abstraction” (Rodaway, 1994, p. 28), however, and as the dominance of the visual is at least partially a cultural construction, a call for more diversity seems justified. This is not only because the other senses deserve more attention, but also because the theories of perception and perceptual memory developed from studies on vision may in fact be theories on *visual* perception and *visual* memory, which do not capture the peculiarities of the other senses (see e.g., Batty, 2011; Barwich, 2019). It has been shown, for example, that “studies of multisensory processing have focused on a narrow region of space in front of the observer” (van der Stoep et al., 2016, p. 513), that is, that even the way non-visual stimuli are presented seems to be biased due to the dominance of vision in research. Thus, basing our theories of perception and perceptual memory mainly on vision may indeed lead to limited and impoverished conceptualizations of perceptual memory. As O’Callaghan (2011) puts it: “Attention to just one sense is bad policy if we’re after a comprehensive and general account of perception, rather than a parochial story about vision” (p. 143).

Second, the necessity of integration: Although I have not explicitly stated this, the ideas presented in the present paper were by and large in line with the Aristotelian view that humans possess five senses. No more than the hierarchy of the senses, however, the number of postulated senses is the same across times and cultures (see e.g., Gold, 1980; Jütte, 2005). Rather, it seems that “[f]rom the invention of the alphabet there has been a continuous drive in the Western world toward the separation of the senses, of functions, of operations, of states emotional and political, as well as of tasks” (McLuhan, 1962, p. 42–43). Given that the different sensory modalities share significant parts of their neural underpinnings, given that the processing of information seems to be rather multimodal than unimodal, and given that our everyday experience is characterized by the concurrent stimulation of our senses, investigating their interactions seems more promising than trying to make more and more fine-grained distinctions (for the attempt to say more about the different kinds of interactions between the senses see e.g., Fulkerson, 2014).

Indeed, it has been shown that the integration and combination of the senses can have an important impact on educational outcomes (see e.g., Keehner and Lowe, 2010; Reid et al., 2019) – not to forget that multisensory integration plays a crucial role in several research areas on high-level cognition, such as the interactions between perception and action or embodied cognition. In the field of embodied cognition, for example, it is believed that “cognition depends upon the kinds of experience that come from having a body with various sensorimotor capacities, and […] that these individual sensorimotor capacities are themselves embedded in a more encompassing biological, psychological, and cultural context” (Varela et al., 1991, p. 173), that is, that cognition cannot be understood without understanding the co-presence of these various sensorimotor capacities (for a similar, early account, see Gibson, 1979; for a more recent perspective on embodied cognition, see Shapiro, 2011).

All in all, investigating the seemingly easy to answer question as to why there is so much more research on vision than on any other sensory modality does not only lead us right into the middle of historical changes and cultural differences, but also gives us the opportunity to take a step back and to start thinking about visual dominance. If the degree to which vision dominates research on the different sensory modalities is not an unchangeable necessity, what kind of sensory environments do we want to create and what kind of research do we want to conduct?

## Ethics Statement

Ethical review and approval was not required for the study on human participants in accordance with the local legislation and institutional requirements. The patients/participants provided their written informed consent to participate in this study.

## Author Contributions

FH developed the research idea and wrote the manuscript.

## Conflict of Interest

The author declares that the research was conducted in the absence of any commercial or financial relationships that could be construed as a potential conflict of interest.

## Footnotes

1 When speaking about “visual memory,” “auditory memory,” etc., I do not simply mean “memory for information acquired through vision/audition,” but memory for the perceptual qualities of a certain stimulation (i.e., perceptual memory).

2 Although the distinction between distant and proximal senses is well established in the literature, one may argue that the distinction is not that strict after all, as one can feel the sun (i.e., a distant stimulus) one one’s back, for instance.

3 This does not imply that we are consciously aware of *all* visual information. As phenomena like change blindness (Rensink et al., 1997) or inattentional amnesia (Simons and Chabris, 1999) demonstrate, we often miss even significant changes in our surroundings. Nevertheless, vision seems to capture a far greater percentage of our attention than the other modalities (for an intuitive rule of thumb, see Heilig, 1992).

4 The idea that the available technology mediates how research questions are investigated, can also be made clear by looking at the history of attention research, for instance: For various theoretical reasons, but also because “multi-channel tape recorders became available at the time and provided an elegant way of presenting stimuli,” nearly all “all the early experiments on attention used auditory” (Styles, 2006, p. 16). This only began to change when computers started to spread all over the world.

5 Just as the “eye-minded philosophy of the Enlightenment” (Classen, 1993, p. 27) changed the way of structuring and organizing gardens, it also changed the way people approached anatomical drawings (Massey, 2017), that is not only the way people thought about the non-visual senses, but also the way they thought about vision itself. Before the 18th century, there had been no apparent conflict between aesthetically appealing and practically useful anatomical drawings of the human body. Anatomical drawings were largely based on the writings of Galen and often contained allegorical depictions to foster the understanding of the human body. One may even say that the anatomical drawings – although being visual, of course – were *not about being visual* in the sense of an accurate depiction of reality, but about combining different aspects ranging from natural philosophy to religion and medicine as well as about activating different senses (see also Borland, 2018). This began to change in the age of Enlightenment: The new norm were accurate drawings based on exact observations, often made during dissections – while artistic depictions of the human body became a clearly separated category of their own. In other words, in the anatomy of the 18th century, the visual became *important as visual*.
